# Effects of dietary intervention on diabetic nephropathy: an umbrella review of systematic reviews and meta-analyses of randomized controlled trials

**DOI:** 10.3389/fendo.2024.1385872

**Published:** 2024-04-29

**Authors:** Linli Cai, Yin Huang, Xingyuan Li, Dehong Cao, Fang Liu

**Affiliations:** ^1^ Department of Nephrology, West China Hospital, Sichuan University, Chengdu, China; ^2^ Department of Urology, West China Hospital, Sichuan University, Chengdu, China; ^3^ Department of Urology, Karamay People’s Hospital of Xinjiang Uygur Autonomous Region, Karamay, China

**Keywords:** diets, DN, umbrella review, meta-analysis, systematic review

## Abstract

**Objective:**

To evaluate the quality of evidence, potential biases, and validity of all available studies on dietary intervention and diabetic nephropathy (DN).

**Methods:**

We conducted an umbrella review of existing meta-analyses of randomized controlled trials (RCTs) that focused on the effects of dietary intervention on DN incidence. The literature was searched via PubMed, Embase, Web of Science, and the Cochrane Database of Systematic Reviews. According to the Grading of Recommendations, Assessment, Development and Evaluation (GRADE), evidence of each outcome was evaluated and graded as “high”, “moderate”, “low” or “very low” quality to draw conclusions. Additionally, we classified evidence of outcomes into 4 categories.

**Results:**

We identified 36 meta-analyses of RCTs and 55 clinical outcomes of DN from 395 unique articles. Moderate-quality evidence suggested that probiotic supplementation could significantly improve blood urea nitrogen (BUN), total cholesterol (TC) and low-density lipoprotein cholesterol (LDL-C) levels in DN patients. Low-quality evidence indicated that probiotic supplementation significantly improved the serum creatinine concentration, urinary albumin–creatinine ratio (UACR), fasting blood glucose (FBG), HbA1c and high-density lipoprotein cholesterol (HDL-C) in DN patients. In addition, low-quality evidence suggested that a salt restriction diet could significantly improve the creatinine clearance rate (CrCl) in patients with DN. Low-quality evidence suggested that vitamin D supplementation could significantly improve the UACR in patients with DN. In addition, low-quality evidence has indicated that soy isoflavone supplementation could significantly improve BUN, FBG, total cholesterol (TC), triglyceride (TG) and LDL-C levels in patients with DN. Furthermore, low-quality evidence suggested that coenzyme Q10 supplementation could significantly improve HbA1c, TC and HDL-C in patients with DN, and dietary polyphenols also significantly improved HbA1c in patients with DN. Finally, low-quality evidence suggested that supplementation with antioxidant vitamins could significantly improve the serum creatinine concentration, systolic blood pressure, and HbA1c level in patients with DN. Given the small sample size, all significantly associated outcomes were evaluated as class IV evidence.

**Conclusion:**

Moderate to low amounts of evidence suggest that supplementation with probiotics, vitamin D, soy isoflavones, coenzyme Q10, dietary polyphenols, antioxidant vitamins, or salt-restricted diets may significantly improve clinical outcomes in patients with DN.

**Systematic Review Registration:**

https://www.crd.york.ac.uk/PROSPERO/, identifier CRD42024512670.

## Introduction

Diabetic nephropathy (DN), a common microvascular complication of diabetes, is an important cause of chronic kidney disease (CKD) and end-stage renal disease. Patients with DN often need dialysis to maintain life, and this condition has a high fatality rate (1). There are many risk factors affecting the occurrence and development of DN, among which the most important risk factors include family history, hypertension, dyslipidemia, obesity and insulin resistance; other risk factors include elevated HbA1c levels, elevated systolic blood pressure, proteinuria and smoking (2). Dietary intervention is an important means to control the progression of DN by reducing the risk factors for DN. The main goal of DN treatment is to prevent microalbuminuria from progressing to macroalbuminuria and ultimately to protect renal function. By controlling a healthy and balanced diet, DN patients can delay the progression of kidney damage and related secondary diseases, such as hypertension, hyperlipidemia, and uremia; in contrast, an unhealthy diet will burden kidney function. Therefore, maintaining a delicate balance between nutrient intake and physiological load is essential for maintaining patients’ quality of life (3).

Effective diet management can not only help control DN but also improve the quality of life of patients (2, 3). According to the current literature, dietary interventions such as probiotic supplementation, a low-salt diet, soy isoflavone supplementation, vitamin supplementation and coenzyme Q10 supplementation can effectively improve the clinical outcome of DN patients, delay the progression of DN, and improve their quality of life (4–11).

Although numerous meta-analyses of randomized controlled trials (RCTs) have evaluated a range of effects of dietary intervention on DN incidence in recent years, drawbacks in terms of the research design, differences in assessments of exposure factors, and inconsistent outcomes have made it difficult to draw definitive conclusions (4–39). Before developing effective dietary management strategies for DN, it is necessary to systematically evaluate the quality of the methodology, potential biases, and validity of all studies available for the effects of dietary intervention on DN. Therefore, we conducted an umbrella review of the meta-analyses to provide an overview of the evidence on the effects of dietary intervention on DN.

## Methods and analysis

### Design and registration

We systematically searched, extracted, and analyzed the data from reported systematic reviews and meta-analyses that focused on the effects of dietary intervention on DN according to the Preferred Reporting Items for Systematic Reviews and Meta-Analysis (PRISMA) guidelines (40). The present umbrella review adhered to the methodological guidance outlined in the Joanna Briggs Institute Manual for Evidence Synthesis of Umbrella Reviews (41) and followed the procedures delineated in the Cochrane Handbook for Conducting Systematic Reviews (42). Furthermore, we proactively enrolled our umbrella review in the International Prospective Register of Systematic Reviews (PROSPERO), with the registration number CRD42024512670. (https://www.crd.york.ac.uk/PROSPERO/).

### Eligibility criteria

Systematic reviews and meta-analyses of RCTs evaluating the effects of dietary intervention on DN incidence in individuals of any ethnicity or sex in all countries and settings were eligible for inclusion. Data on individual dietary interventions were extracted separately if two or more dietary interventions were reported in a single meta-analysis. If two or more meta-analyses (those published more than 24 months apart) were performed on the same dietary intervention and clinical outcome of DN, we included the latest meta-analysis for data analysis. In the event that multiple meta-analyses were conducted within a 24-month timeframe, preference was given to the meta-analysis encompassing the highest number of RCTs. If an equal number of RCTs existed, priority was assigned to the meta-analysis with a superior AMSTAR score. In addition, if the latest meta-analysis did not perform dose-response analysis, while another meta-analysis did, both studies were included for data extraction. Non-English studies and animal and cell culture studies were also excluded.

### Population

This umbrella review is centered on systematically reviewing meta-analyses that assess the effects of dietary intervention on DN. The primary focus of the original articles incorporated within these systematic reviews and meta-analyses should be directed toward identifying dietary interventions that have the potential to either improve or exacerbate the clinical outcomes of DN. Studies evaluating the efficacy of a certain dietary intervention for the risk of DN were excluded.

### Exposure

We included a meta-analysis that reported at least 1 type of dietary intervention for DN, including probiotics, a salt restriction diet, vitamin D, soy isoflavone, and low-protein diets. The efficacy of dietary intervention on the clinical outcomes of DN was evaluated by the risk ratio (RR), mean difference (MD) or standard mean difference (SMD) with 95% confidence intervals (CIs).

### Outcomes

The outcomes of this umbrella review included endocrine metabolic outcomes, including the urinary albumin excretion rate (UAER), serum creatinine (Scr), blood urea nitrogen (BUN), the urinary albumin–creatinine ratio (UACR), fasting blood glucose (FBG), total cholesterol (TC), triglycerides (TG), low-density lipoprotein cholesterol (LDL-C), high-density lipoprotein cholesterol (HDL-C), systolic blood pressure (SBP), diastolic blood pressure (DBP), coenzyme Q10 (CoQ10), the glomerular filtration rate (GFR), and the creatinine clearance rate (CrCl).

### Study designs

Only systematic reviews and meta-analyses of RCTs evaluating the effects of dietary intervention on DN incidence in individuals of any ethnicity or sex in all countries and settings were eligible for inclusion. All the included systematic reviews and meta-analyses needed to focus on dietary intervention in DN patients and describe the meta-analysis methods in detail, including the complete search strategy, inclusion and exclusion criteria, literature quality evaluation criteria, result evaluation methods, analysis methods and procedures, and interpretation criteria.

### Information sources

In our study, we systematically searched PubMed, Embase, the Web of Science, and the Cochrane Database of Systematic Reviews until July 2023 for relevant systematic reviews and meta-analyses of RCTs. We also reviewed the reference lists of the included meta-analyses to find additional relevant articles.

### Search strategy

The databases were accessed using Medical Subject Headings (MeSH), keywords, and text terms related to dietary intervention and DN, following the Scottish Intercollegiate Guidelines Network (SIGN) recommendations for literature search methodology (43). The detailed search strategy for PubMed was as follows: (((“Diabetic Nephropathies”[Mesh]) OR (((((((((((((((((Nephropathies, Diabetic) OR (Nephropathy, Diabetic)) OR (Diabetic nephropathy)) OR (Diabetic Kidney Disease)) OR (Diabetic Kidney Diseases)) OR (Kidney Disease, Diabetic)) OR (Kidney Diseases, Diabetic)) OR (Diabetic Glomerulosclerosis)) OR (Glomerulosclerosis, Diabetic)) OR (Intracapillary Glomerulosclerosis)) OR (Nodular Glomerulosclerosis)) OR (Glomerulosclerosis, Nodular)) OR (Kimmelstiel-Wilson Syndrome)) OR (Kimmelstiel Wilson Syndrome)) OR (Syndrome, Kimmelstiel-Wilson)) OR (Kimmelstiel-Wilson Disease)) OR (Kimmelstiel Wilson Disease))) AND (((“Diet”[Mesh]) OR (diets)) OR ((“Food”[Mesh]) OR (foods)))) AND (meta-analysis OR systematic review).

### Study selection

**Figure 1:**
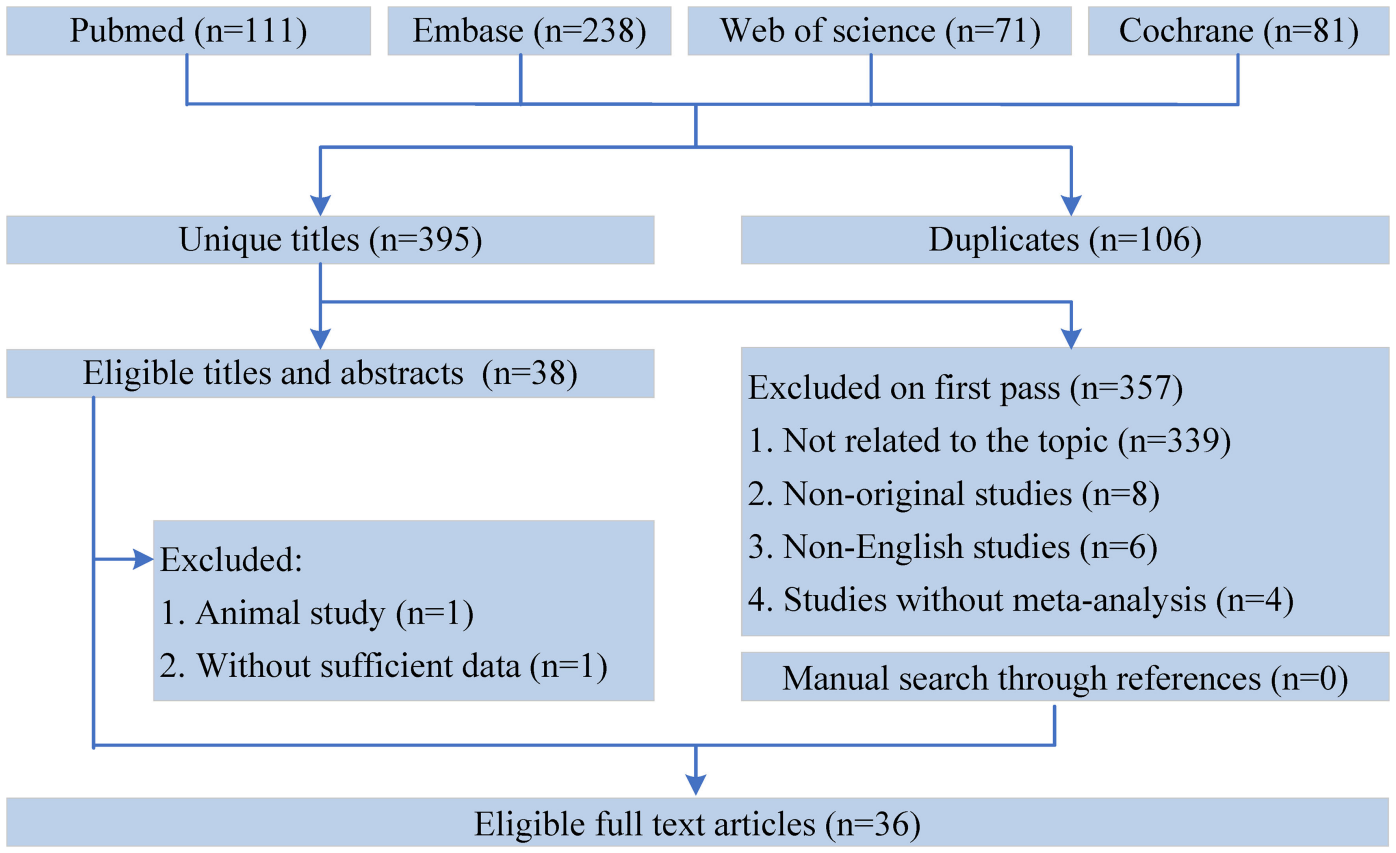
Flowchart of the systematic search and selection process.

All the retrieved literature was screened using Endnote X9. After excluding duplicates, two authors screened the titles and abstracts and identified meta-analyses that met the inclusion criteria through full-text reading independently. All disagreements between the two authors were resolved by a third author. In addition, we hand-searched studies from the reference lists to identify meta-analyses that might have been excluded (**Figure 1**).

### Assessment of methodological quality

The methodological quality of each meta-analysis was assessed by two authors using AMSTAR, a validated, stringent, and reliable tool for evaluating systematic reviews and meta-analyses (44, 45). In addition, according to the Grading of Recommendations, Assessment, Development and Evaluation (GRADE), evidence of each health outcome was evaluated and graded as “high”, “moderate”, “low” or “very low” quality to draw conclusions (46). Additionally, we classified the evidence of outcomes into 4 categories following the evidence classification criteria: class I (convincing evidence), class II (highly suggestive evidence), class III (suggestive evidence), class IV (weak evidence) and NS (nonsignificant) (47–50). The detailed criteria for evidence classification are shown in **Table 1**.

**Table 1:** Evidence categories criteria.

Evidence class	Description
Class I: convincing evidence	>1000 cases (or >20,000 participants for continuous outcomes), statistical significance at *P* < 10^−6^ (random-effects), no evidence of small-study effects and excess significance bias; 95% prediction interval excluded the null, no large heterogeneity (I^2^ < 50%)
Class II: highly suggestive evidence	>1000 cases (or >20,000 participants for continuous outcomes), statistical significance at *P* < 10^−6^ (random-effects) and largest study with 95% CI excluding the null value
Class III: suggestive evidence	>1000 cases (or >20,000 participants for continuous outcomes) and statistical significance at *P* < 0.001
Class IV: weak evidence	The remaining significant associations with *P* < 0.05
NS: non-significant	*P* > 0.05

### Data extraction

Two investigators autonomously retrieved the pertinent data from each qualifying study: 1) name of the author, 2) publication date, 3) dietary intervention, 4) control, 5) outcomes, 6) number of included studies, 7) sample size, 8) length of follow-up, and 9) MD or SMD estimates with 95% CIs. In addition, we extracted the meta-analytic model used (random or fixed), estimate of heterogeneity (*I* ^2^ and Cochran’s *Q* test) and small-study assessment (Egger’s test, Begg’s test and funnel plot). When dose response analysis and subgroup analysis were performed, we extracted the *P* value for nonlinearity and any reported estimate for subgroup analysis. Any disagreements were resolved by a third author.

### Data summary

We recalculated the RR, MD or SMD with 95% CIs through random or fixed effects models and evaluated the heterogeneity (*I* ^2^ and Cochran’s *Q* test) and small-study effects (Egger or Begg test for each systematic review and meta-analysis with more than 10 studies) in each meta-analysis when sufficient data were provided (51–53). For dietary interventions identified as class I-II evidence, high-quality evidence or moderate-quality evidence, we conducted sensitivity analysis when sufficient data were available to determine the effect of some individual studies on the total significance of the evidence. Dose-response analysis of DN incidence associated with any dietary intervention was also performed. Furthermore, if the most recent meta-analysis did not involve clinical studies that involved other meta-analyses, we combined the data of these studies and performed a reanalysis. A *P* < 0.10 was considered to indicate heterogeneity, and for other tests, *P* < 0.05 was considered to indicate statistical significance. Review Manager v5.4.1 (Cochrane Collaboration, Oxford, UK) was used for evidence synthesis. Egger and Begg tests, along with sensitivity analysis, were performed using Stata v15.1.

## Major outcomes

### Characteristics of the meta-analyses

**Figure 2:**
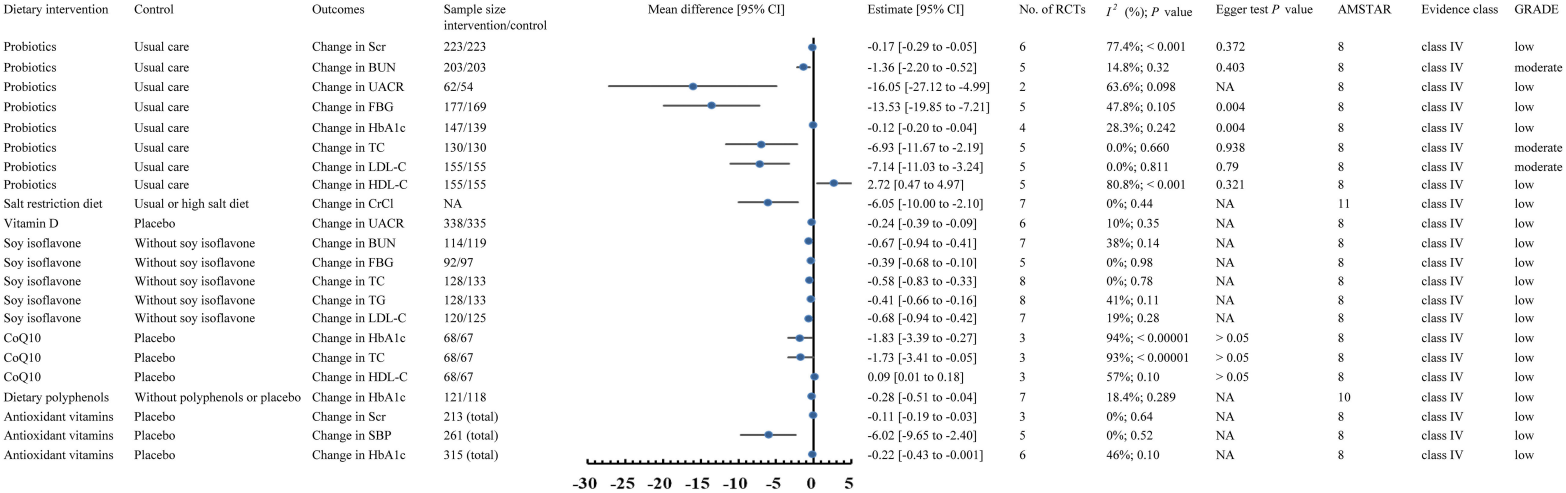
Forest plots of moderate-quality evidence and low-quality evidence for dietary interventions that could significantly improve clinical outcomes in patients with DN. Scr, serum creatinine; BUN, blood urea nitrogen; UACR, urinary albumin creatinine ratio; FBG, fasting blood-glucose; TC, total cholesterol; TG, triglycerides; LDC-L, low-density lipoprotein cholesterol; HDL-C, high-density lipoprotein cholesterol; SBP, systolic blood pressure; CoQ10, coenzyme Q10; CrCl, Creatinine clearance rate; RCT, randomized controlled trial; MD, mean difference; AMSTAR, a measurement tool to assess systematic reviews; GRADE, Grading of Recommendations Assessment, Development, and Evaluation; NA, not available.

A flowchart of the literature search and selection process is presented in **Figure 1**. After a systematic literature search, 501 unique articles were identified. A total of 36 meta-analyses were yielded based on our inclusion criteria. We extracted 9 unique dietary interventions (including probiotics, a salt restriction diet, vitamin D, soy isoflavone, CoQ10, ketoanalog, dietary polyphenols, antioxidant vitamins, and low-protein diets) and 55 corresponding outcomes in meta-analyses, including 34 significantly associated outcomes and 21 nonsignificantly associated outcomes (**Table 2**). After a careful evaluation of evidence quality using established criteria, all outcomes were classified as IV or NS (nonsignificant) evidence. In addition, according to the GRADE rating criteria, only five dietary interventions were rated as moderate-quality evidence, 33 were rated as low-quality evidence, and 17 were rated as very low-quality evidence (**Table 2**). Moderate-quality evidence and low-quality evidence for dietary interventions that could significantly improve clinical outcomes in patients with DN are presented in **Figure 2**.

**Table 2:** Effects of dietary intervention on diabetic nephropathy.

Dietary intervention	Control	Outcomes	Total eligible MA	Included MA	Sample size intervention/control	MA metric	Estimates [95% CI]	No. of RCTs	Effects model	*I* ^2^; *Q* test *P* value	Egger test *P* value	AMSTAR	Evidence class	GRADE
Significant associations														
Low protein diets	Usual diet	Change in UAER	19	Jiang 2023	22/23	SMD	0.68 [0.08 to 1.29]	2	Random	0%; 0.81	NA	11	class IV	very low
Probiotics	Usual care	Change in Scr	5	Dai 2022	223/223	MD	-0.17 [-0.29 to -0.05]	6	Random	77.4%; < 0.001	0.372	8	class IV	low
Probiotics	Usual care	Change in BUN	5	Dai 2022	203/203	MD	-1.36 [-2.20 to -0.52]	5	Random	14.8%; 0.32	0.403	8	class IV	moderate
Probiotics	Usual care	Change in UACR	5	Dai 2022	62/54	MD	-16.05 [-27.12 to -4.99]	2	Random	63.6%; 0.098	NA	8	class IV	low
Probiotics	Usual care	Change in FBG	5	Dai 2022	177/169	MD	-13.53 [-19.85 to -7.21]	5	Random	47.8%; 0.105	0.004	8	class IV	low
Probiotics	Usual care	Change in HbA1c	5	Dai 2022	147/139	MD	-0.12 [-0.20 to -0.04]	4	Random	28.3%; 0.242	0.004	8	class IV	low
Probiotics	Usual care	Change in TC	5	Dai 2022	130/130	MD	-6.93 [-11.67 to -2.19]	5	Random	0.0%; 0.660	0.938	8	class IV	moderate
Probiotics	Usual care	Change in LDL-C	5	Dai 2022	155/155	MD	-7.14 [-11.03 to -3.24]	5	Random	0.0%; 0.811	0.790	8	class IV	moderate
Probiotics	Usual care	Change in HDL-C	5	Dai 2022	155/155	MD	2.72 [0.47 to 4.97]	5	Random	80.8%; < 0.001	0.321	8	class IV	low
Salt restriction diet	Usual or high salt diet	Change in SBP	3	Hodson 2023	400 (total)	MD	-7.36 [-10.75 to -3.98]	12	Random	74%; < 0.0001	NA	11	class IV	very low
Salt restriction diet	Usual or high salt diet	Change in DBP	3	Hodson 2023	400 (total)	MD	-3.17 [-4.58 to -1.76]	12	Random	54%; 0.01	NA	11	class IV	very low
Salt restriction diet	Usual or high salt diet	Change in CrCl	3	Hodson 2023	NA	MD	-6.05 [-10.00 to -2.10]	7	Random	0%; 0.44	NA	11	class IV	low
Salt restriction diet	Usual or high salt diet	Change in body weight	3	Hodson 2023	NA	MD	-1.21 [-1.73 to -0.68]	12	Random	76%; < 0.00001	NA	11	class IV	very low
Vitamin D	Placebo	Change in UACR	4	He 2022	338/335	SMD	-0.24 [-0.39 to -0.09]	6	Fixed	10%; 0.35	NA	8	class IV	low
Vitamin D	Placebo	Change in UAER	4	He 2022	477/397	SMD	-0.42 [-0.53 to -0.32]	4	Fixed	55%; 0.02	NA	8	class IV	very low
Vitamin D	Without vitamin D or placebo	Change in 24-hour urine protein	4	Wang 2019	409/407	MD	-0.26 [-0.34 to -0.17]	11	Random	95%; < 0.00001	NA	8	class IV	very low
Soy isoflavone	Without soy isoflavone	Change in 24-hour urine protein	1	Wang 2021	114/111	SMD	-2.58 [-3.94 to -1.22]	6	Random	93%; < 0.00001	NA	8	class IV	very low
Soy isoflavone	Without soy isoflavone	Change in BUN	1	Wang 2021	114/119	SMD	-0.67 [-0.94 to -0.41]	7	Fixed	38%; 0.14	NA	8	class IV	low
Soy isoflavone	Without soy isoflavone	Change in FBG	1	Wang 2021	92/97	SMD	-0.39 [-0.68 to -0.10]	5	Fixed	0%; 0.98	NA	8	class IV	low
Soy isoflavone	Without soy isoflavone	Change in TC	1	Wang 2021	128/133	SMD	-0.58 [-0.83 to -0.33]	8	Fixed	0%; 0.78	NA	8	class IV	low
Soy isoflavone	Without soy isoflavone	Change in TG	1	Wang 2021	128/133	SMD	-0.41 [-0.66 to -0.16]	8	Fixed	41%; 0.11	NA	8	class IV	low
Soy isoflavone	Without soy isoflavone	Change in LDL-C	1	Wang 2021	120/125	SMD	-0.68 [-0.94 to -0.42]	7	Fixed	19%; 0.28	NA	8	class IV	low
CoQ10	Placebo	Change in FBG	1	Zhang 2019	68/67	SMD	-2.04 [-3.90 to -0.18]	3	Random	93%; < 0.00001	> 0.05	8	class IV	very low
CoQ10	Placebo	Change in HbA1c	1	Zhang 2019	68/67	MD	-1.83 [-3.39 to -0.27]	3	Random	94%; < 0.00001	> 0.05	8	class IV	low
CoQ10	Placebo	Change in TC	1	Zhang 2019	68/67	SMD	-1.73 [-3.41 to -0.05]	3	Random	93%; < 0.00001	> 0.05	8	class IV	low
CoQ10	Placebo	Change in HDL-C	1	Zhang 2019	68/67	MD	0.09 [0.01 to 0.18]	3	Random	57%; 0.10	> 0.05	8	class IV	low
Ketoanalogue	Without ketoanalogue	Change in 24-hour urine protein	1	Bellizzi 2022	246 (total)	MD	-1.41 [-2.74 to -0.08]	5	Random	99.42%; < 0.001	NA	8	class IV	very low
Ketoanalogue	Without ketoanalogue	Change in FBG	1	Bellizzi 2022	310 (total)	MD	-27.57 [-39.20 to -15.94]	7	Random	96.7%; < 0.001	NA	8	class IV	very low
Dietary polyphenols	Without polyphenols or placebo	Change in HbA1c	1	Macena 2022	121/118	MD	-0.28 [-0.51 to -0.04]	7	Random	18.4%; 0.289	NA	10	class IV	low
Dietary polyphenols	Without polyphenols or placebo	Change in GFR	1	Macena 2022	170/163	MD	3.66 [0.16 to 7.15]	7	Random	59.7%; 0.021	NA	10	class IV	very low
Dietary polyphenols	Without polyphenols or placebo	Change in 24-hour urine protein	1	Macena 2022	96/93	MD	-109.10 [-216.57 to -1.63]	5	Random	86.8%; < 0.001	NA	10	class IV	very low
Antioxidant vitamins	Placebo	Change in Scr	1	Chen 2020	213 (total)	MD	-0.11 [-0.19 to -0.03]	3	Random	0%; 0.64	NA	8	class IV	low
Antioxidant vitamins	Placebo	Change in SBP	1	Chen 2020	261 (total)	MD	-6.02 [-9.65 to -2.40]	5	Fixed	0%; 0.52	NA	8	class IV	low
Antioxidant vitamins	Placebo	Change in HbA1c	1	Chen 2020	315 (total)	MD	-0.22 [-0.43 to -0.001]	6	Fixed	46%; 0.10	NA	8	class IV	low
*Non-significant associations*														
Low protein diets	Usual diet	All-cause mortality	19	Jiang 2023	180/178	RR	0.38 [0.10 to 1.44]	5	Random	0%; 0.43	NA	11	NS	low
Low protein diets	Usual diet	Renal failure	19	Jiang 2023	141/146	RR	1.16 [0.38 to 3.59]	4	Random	0%; 0.79	NA	11	NS	low
Low protein diets	Usual diet	Change in GFR	19	Jiang 2023	189/178	MD	-0.73 [-2.30 to 0.83]	7	Random	53%; 0.05	NA	11	NS	very low
Low protein diets	Usual diet	Change in CrCl	19	Jiang 2023	107/96	MD	-2.39 [-5.87 to 1.08]	3	Random	53%; 0.12	NA	11	NS	very low
Low protein diets	Usual diet	Change in 24-hour urinary albumin excretion	19	Jiang 2023	60/59	MD	0.00 [-0.07 to 0.07]	2	Random	0%; 0.75	NA	11	NS	very low
Probiotics	Placebo	Change in GFR	5	Dai 2022	190/182	MD	4.51 [-0.03 to 9.06]	5	Random	87.0%; < 0.001	0.155	8	NS	low
Salt restriction diet	Usual or high salt diet	Change in GFR	3	Hodson 2023	NA	MD	-1.87 [-5.05 to 1.31]	12	Random	32%; 0.13	NA	11	NS	low
Salt restriction diet	Usual or high salt diet	Change in HbA1c	3	Hodson 2023	NA	MD	-0.62 [-1.49 to 0.26]	6	Random	95%; < 0.00001	NA	11	NS	very low
Vitamin D	Without vitamin D or placebo	Change in Scr	4	Wang 2019	283/277	MD	-0.83 [-3.67 to 2.02]	9	Fixed	0%; 0.95	NA	8	NS	low
Vitamin D	Without vitamin D or placebo	Change in GFR	4	Wang 2019	147/143	MD	2.13 [-2.06 to 6.32]	4	Fixed	0%; 0.82	NA	8	NS	low
Vitamin D	Without vitamin D or placebo	Change in HbA1c	4	Wang 2019	348/344	MD	0.01 [-0.09 to 0.11]	10	Random	0%; 0.72	NA	8	NS	moderate
Vitamin D	Without vitamin D or placebo	Change in FBG	4	Wang 2019	115/115	MD	-0.05 [-0.29 to 0.20]	3	Fixed	0%; 0.78	NA	8	NS	low
Soy isoflavone	Without soy isoflavone	Change in body weight	1	Wang 2021	106/111	SMD	-0.05 [-0.32 to 0.21]	6	Fixed	0%; 1.00	NA	8	NS	low
Soy isoflavone	Without soy isoflavone	Change in Scr	1	Wang 2021	120/125	SMD	-0.24 [-0.49 to 0.01]	7	Fixed	0%; 0.93	NA	8	NS	low
Soy isoflavone	Without soy isoflavone	Change in CrCl	1	Wang 2021	36/36	SMD	-0.36 [-0.83 to 0.10]	3	Fixed	0%; 0.46	NA	8	NS	low
Soy isoflavone	Without soy isoflavone	Change in GFR	1	Wang 2021	100/105	SMD	-0.07 [-0.35 to 0.20]	6	Fixed	0%; 0.79	NA	8	NS	low
Soy isoflavone	Without soy isoflavone	Change in HDL-C	1	Wang 2021	120/125	SMD	0.16 [-0.09 to 0.41]	7	Fixed	0%; 0.64	NA	8	NS	low
CoQ10	Placebo	Change in LDL-C	1	Zhang 2019	68/67	SMD	-0.27 [-0.62 to 0.07]	3	Fixed	0%; 0.50	> 0.05	8	NS	moderate
Ketoanalogue	Without ketoanalogue	Change in GFR	1	Bellizzi 2022	221 (total)	MD	4.06 [-1.84 to 9.97]	4	Random	99.42%; < 0.001	NA	8	NS	very low
Antioxidant vitamins	Placebo	Change in DBP	1	Chen 2020	261 (total)	MD	-1.19 [-3.91 to 1.52]	5	Fixed	0%; 0.85	NA	8	NS	low
Antioxidant vitamins	Placebo	Change in FBG	1	Chen 2020	222 (total)	MD	-1.12 [-13.24 to 10.99]	5	Fixed	0%; 0.58	NA	8	NS	low

MA, meta-analysis; CI, confidence interval; UAER, Urinary albumin excretion rate; Scr, serum creatinine; BUN, blood urea nitrogen; UACR, urinary albumin creatinine ratio; FBG, fasting blood-glucose; TC, total cholesterol; TG, triglycerides; LDL-C, low-density lipoprotein cholesterol; HDL-C, high-density lipoprotein cholesterol; SBP, systolic blood pressure; DBP, diastolic blood pressure; CoQ10, coenzyme Q10; GFR, glomerular filtration rate; CrCl, creatinine clearance rate; RCT, randomized controlled trial; RR, risk ratio; MD, mean difference; SMD, standard mean difference; NA, not available.

### Probiotics

A total of 5 meta-analyses (11, 16, 17, 19, 21) studied the efficacy of probiotic intervention for DN. The meta-analysis published by Dai et al. in 2022 (16) included 6 RCTs describing 446 patients with DN, in which Lactobacillus acidophilus, Lactobacillus casei, Lactobacillus lactis, Bifidobacterium bifidum, Bifidobacterium longum, Bifidobacterium infants, Lactobacillus plantarum A7, Lactobacillus fermentum strain ZT-L3, Bacillus coagulans T11, and Streptococcus thermophilus were included for pooled analysis. An umbrella review found that probiotic intervention could significantly improve LDL-C (MD -7.14, 95% CI -11.03 to -3.24) (moderate-quality evidence), TC (MD -6.93, 95% CI -11.67 to -2.19) (moderate-quality evidence), BUN (MD -1.36, 95% CI -2.20 to -0.52) (moderate-quality evidence), Scr (MD -0.17, 95% CI -0.29 to -0.05) (low-quality evidence), UACR (MD -16.05, 95% CI -27.12 to -4.99) (low-quality evidence), FBG (MD -13.53, 95% CI -19.85 to -7.21) (low-quality evidence), HbA1c (MD -0.12, 95% CI -0.20 to -0.04) (low-quality evidence), and HDL-C (MD 2.72, 95% CI 0.47 to 4.97) (low-quality evidence) in DN patients compared with conventional care without probiotics. However, an umbrella review revealed that probiotic intervention had no significant improvement on the glomerular filtration rate (GFR) (MD 4.51, 95% CI -0.03 to 9.06) (low-quality evidence) in DN patients (**Figure 2**) (**Table 2**).

### Salt restriction diet

A total of 3 meta-analyses (10, 13, 30) studied the effect of a salt restriction diet on DN incidence. The meta-analysis of Hodson et al. published in 2023 (13) included 12 RCTs describing 400 patients with DN. An umbrella review revealed that, compared with a usual or high-salt diet, a salt restriction diet significantly improved SBP (MD -7.36, 95% CI -10.75 to -3.98) (very low-quality evidence), DBP (MD -3.17, 95% CI -4.58 to -1.76) (very low-quality evidence), CrCl (MD -6.05, 95% CI -10.00 to -2.10) (low-quality evidence), and body weight (MD -1.21, 95% CI -1.73 to -0.68) (very low-quality evidence) in DN patients. However, an umbrella review revealed that a salt restriction diet had no significant improvement on the glomerular filtration rate (GFR) (MD -1.87, 95% CI -5.05 to 1.31) (low-quality evidence) or HbA1c (SMD -0.62, 95% CI -1.49 to 0.26) (very low-quality evidence) in DN patients (**Figure 2**) (**Table 2**).

### Soy isoflavone

A total of 1 meta-analysis studied the effect of soy isoflavone supplementation on DN incidence. The meta-analysis of Wang et al. published in 2021 (5) included eight RCTs involving 261 patients with DN. An umbrella review revealed that, compared with no supplementation with soy isoflavones, supplementation with soy isoflavones significantly improved BUN (SMD -0.67, 95% CI -0.94 to -0.41) (low-quality evidence), FBG (SMD -0.39, 95% CI -0.68 to -0.10) (low-quality evidence), total cholesterol (TC) (SMD -0.58, 95% CI -0.83 to -0.33) (low-quality evidence), total glucose (TG) (SMD -0.41, 95% CI -0.66 to -0.16) (low-quality evidence), LDL-C (SMD -0.68, 95% CI -0.94 to -0.42) (low-quality evidence) and 24-hour urine protein (SMD -2.58, 95% CI -3.94 to -1.22) (very low-quality evidence) in DN patients. However, an umbrella review revealed that supplementation with soy isoflavones had no significant improvement on body weight (SMD -0.05, 95% CI -0.32 to 0.21; low-quality evidence), Scr (SMD -0.24, 95% CI -0.49 to 0.01; low-quality evidence), CrCl (SMD -0.36, 95% CI -0.83 to 0.10; low-quality evidence), GFR (SMD -0.07, 95% CI -0.35 to 0.20; low-quality evidence) or HDL-C (SMD 0.16, 95% CI -0.09 to 0.41; low-quality evidence) in DN patients (**Figure 2**) (**Table 2**).

### Vitamin

A total of 4 meta-analyses (4, 22, 24, 28) studied the effect of vitamin D supplementation on DN incidence. The meta-analysis of He et al. published in 2022 (4) included 6 RCTs involving 874 patients with DN. A review of Umbrella medicine showed that, compared with placebo, vitamin D supplementation significantly improved the UACR (SMD -0.24, 95% CI -0.39 to -0.09) (low-quality evidence), UAER (SMD -0.42, 95% CI -0.53 to -0.32) (very low-quality evidence), and 24-hour urine protein (MD -0.26, 95% CI -0.34 to -0.17) (very low-quality evidence) in DN patients. However, an umbrella review of the meta-analysis of Wang et al. published in 2019 (22) revealed that vitamin D supplementation had no significant improvement on Scr (MD -0.83, 95% CI -3.67 to 2.02) (low-quality evidence), GFR (MD 2.13, 95% CI -2.06 to 6.32) (low-quality evidence), HbA1c (MD 0.01, 95% CI -0.09 to 0.11) (moderate-quality evidence), or FBG (MD -0.05, 95% CI -0.29 to 0.20) (low-quality evidence) in DN patients (**Figure 2**) (**Table 2**).

In addition, a total of 1 meta-analysis studied the effect of antioxidant vitamin supplementation on DN. The meta-analysis of Chen et al. published in 2020 (9) included 6 RCTs involving 315 patients with DN. An umbrella review revealed that, compared with placebo, supplementation with antioxidant vitamins significantly improved Scr (MD -0.11, 95% CI -0.19 to -0.03) (low-quality evidence), SBP (MD -6.02, 95% CI -9.65 to -2.40) (low-quality evidence), and HbA1c (MD -0.22, 95% CI -0.43 to -0.001) (low-quality evidence) in DN patients. However, an umbrella review revealed that supplementation with antioxidant vitamins did not significantly improve DBP (MD -1.19, 95% CI -3.91 to 1.52) (low-quality evidence) or FBG (MD -1.12, 95% CI -13.24 to 10.99) (low-quality evidence) in DN patients (**Figure 2**) (**Table 2**).

### CoQ10

A total of 1 meta-analysis studied the effect of CoQ10 supplementation on DN incidence. The meta-analysis of Zhang et al. published in 2019 (7) included 3 RCTs involving 135 patients with DN. An umbrella review revealed that, compared with placebo, supplementation with CoQ10 significantly improved FBG (SMD -2.04, 95% CI -3.90 to -0.18) (very low-quality evidence), HbA1c (MD -1.83, 95% CI -3.39 to -0.27) (low-quality evidence), total cholesterol (TC) (SMD -1.73, 95% CI -3.41 to -0.05) (low-quality evidence), and high-density lipoprotein cholesterol (HDL-C) (MD 0.09, 95% CI 0.01 to 0.18) (low-quality evidence) in DN patients. However, an umbrella review revealed that supplementation with CoQ10 did not significantly improve LDL-C levels (SMD -0.27, 95% CI -0.62 to 0.07) (moderate-quality evidence) in DN patients (**Figure 2**) (**Table 2**).

### Low-protein diets

A total of 19 meta-analyses (6, 12, 15, 18, 20, 23, 25–27, 29, 31–39) studied the effect of low-protein diets on DN incidence. The meta-analysis of Jiang et al. published in 2023 (12) included 7 RCTs and included 367 patients with DN. An umbrella review revealed that low-protein diets significantly improved the UAER (standardized mean difference (SMD) of 0.68, 95% CI of 0.08 to 1.29) (very low-quality evidence) in DN patients compared with the usual diet. However, the umbrella review revealed that low-protein diets had no significant improvement on all-cause mortality (RR 0.38, 95% CI 0.10 to 1.44) (low-quality evidence), renal failure (RR 1.16, 95% CI 0.38 to 3.59) (low-quality evidence), GFR (MD -0.73, 95% CI -2.30 to 0.83) (very low-quality evidence), CrCl (MD -2.39, 95% CI -5.87 to 1.08) (very low-quality evidence), or 24-hour urinary albumin excretion (MD 0.00, 95% CI -0.07 to 0.07) (very low-quality evidence) in DN patients (**Figure 2**) (**Table 2**).

### Dietary polyphenols

A total of 1 meta-analysis studied the effect of dietary polyphenol supplementation on DN incidence. The meta-analysis of Macena et al. published in 2022 (14) included 7 RCTs describing 333 patients with DN. An umbrella review revealed that dietary polyphenol supplementation significantly improved HbA1c levels (MD -0.28, 95% CI -0.51 to -0.04) (low-quality evidence), glomerular filtration rate (GFR) (MD 3.66, 95% CI 0.16 to 7.15) (very low-quality evidence) and 24-hour urine protein levels (MD -109.10, 95% CI -216.57 to -1.63) (very low-quality evidence) in DN patients compared with those in patients receiving no polyphenols or placebo (**Figure 2**) (**Table 2**).

### Ketoanalog

A total of 1 meta-analysis studied the effect of ketoanalogue supplementation on DN incidence. The meta-analysis of Bellizzi et al. published in 2022 (8) included 7 RCTs of 310 patients with DN. An umbrella review revealed that, compared with the usual diet, ketoanalog supplementation significantly improved 24-hour urine protein (MD -1.41, 95% CI -2.74 to -0.08) (very low-quality evidence) and fasting blood glucose (FBG) (MD -27.57, 95% CI -39.20 to -15.94) (very low-quality evidence) in DN patients. However, an umbrella review revealed that ketoanalogue supplementation had no significant improvement on the glomerular filtration rate (GFR) (MD 4.06, 95% CI -1.84 to 9.97) (very low-quality evidence) in DN patients (**Figure 2**) (**Table 2**).

### Heterogeneity

In our study, 74.5% of the outcomes were reanalyzed using a random or fixed effects model. The reanalysis revealed that approximately 36.6% of the examined outcomes exhibited significant heterogeneity (*I* ^2^ > 50% or Cochran’s Q test *P* < 0.1). The heterogeneity of most of the outcomes could be attributed to various potential factors, such as study setting, geographical region, ethnicity, sex, age, study quality, sample size, follow-up duration, and adjustment for confounding variables. For the remaining 25.5% of the unanalyzed outcomes, approximately 50% exhibited significant heterogeneity.

### Assessment of risk of bias

In our reanalysis, Egger’s test assessed publication bias for 19.5% of the total outcomes, revealing publication bias in 1 of them. For nonreanalyzed outcomes, publication bias was detected in 35.7% of the outcomes via statistical tests or funnel plots. Importantly, other outcomes either showed no significant publication bias or lacked reported bias assessments.

### AMSTAR score, GRADE and evidence classification

The median AMSTAR score for all outcomes was 8 (8-11), and further detailed AMSTAR scores specific to each outcome can be found in **Supplementary Table S1**. For the GRADE, five outcomes (change in BUN (probiotics), change in TC (probiotics), change in LDL-C (probiotics), change in HbA1c (vitamin D), change in LDL-C (CoQ10)) were downgraded to “moderate” quality given the imprecision, and the remaining outcomes were downgraded to “low” or “very low” due to the risk of bias, inconsistency, indirectness, or imprecision. **Supplementary Table S2** shows the detailed GRADE classification for each outcome. In terms of evidence, all outcomes were classified as IV or NS (nonsignificant) because of the small sample size.

## Discussion

### Principal findings and possible explanations

Relevant studies have shown that the incidence of DN is increasing rapidly, and patients with DN accounted for 20% to 40% of type 2 diabetes patients in the community from 2009 to 2012 (54). It is not only the main cause of death in type 1 diabetes patients but also an important factor threatening the health of type 2 diabetes patients (55). At present, it is believed that the disease progression of DN is difficult to reverse, the risk factors involved in the progression of DN cannot be identified, and effective measures cannot be taken to delay the progression of disease to end-stage nephropathy (56). With the increase in the number of DN patients, the disease burden on society and families will also increase (57). In recent years, due to the deepening of basic research, the treatment of DN has taken a new direction. Several scholars have proposed that probiotics may improve and prevent metabolic diseases such as DN through changes in the human intestinal flora (58). In addition, some animal model studies have shown that soy foods can prevent kidney disease and delay the deterioration of kidney function (59, 60). Giving soy foods instead of meat to DN patients can improve kidney function (61, 62). Furthermore, a large number of animal and cellular experiments and clinical studies have shown that active vitamin D has a renoprotective effect and may play a role in inhibiting the inflammatory response, antioxidative stress, and renal fibrosis; inhibiting the renin-angiotensin system; and improving insulin resistance (4, 22, 24, 28).

To date, a large number of researchers worldwide have carried out clinical research and evidence-based medical research on the effects of dietary intervention on DN. This umbrella evaluation evaluated the advantages and disadvantages of existing evidence-based medical methods from systematic reviews and meta-analyses on the effects of dietary intervention on DN, helped us to understand the potential effective dietary management strategies for the prevention and treatment of DN in a more comprehensive way from multiple dimensions, provided a theoretical basis for the development of more clinically effective prevention and control measures for DN, and provided directions for further clinical research.

The present umbrella review extracted 9 unique dietary interventions (including probiotics, a salt restriction diet, vitamin D, soy isoflavone, CoQ10, ketoanalog, dietary polyphenols, antioxidant vitamins, and low-protein diets) and 55 corresponding outcomes in meta-analyses, including 34 significantly associated outcomes and 21 nonsignificantly associated outcomes. All outcomes were classified as IV or NS (nonsignificant), and only five dietary interventions were rated as moderate-quality evidence.

First, compared with conventional care without probiotics, probiotic intervention significantly improved LDL-C (moderate-quality evidence), TC (moderate-quality evidence), BUN (moderate-quality evidence), Scr (low-quality evidence), UACR (low-quality evidence), FBG (low-quality evidence), HbA1c (low-quality evidence), and HDL-C (low-quality evidence) in DN patients. He et al. (63) reported that probiotic supplementation can reduce the abundance of conditioned pathogenic bacteria, increase the abundance of beneficial intestinal bacteria, and reduce the release of enterogenic endotoxin, thus effectively improving blood sugar and blood lipid levels and kidney function. In recent years, an increasing number of studies have shown that inflammatory factors play a certain role in the pathogenesis of DN. Inflammation in DN patients is characterized by increased expression of inflammatory factors, inflammatory chemokines and adhesion factors; inflammatory cell infiltration; and increased CRP levels. Compared with that of classical inflammation, the severity of DN is mild, and DN is associated with a state of microinflammation (64). Firouzi et al. (65) showed that probiotic supplementation could reduce the content of enteric-borne urotoxins (such as para-cresol and indoxyl sulfate) in the blood of DN patients, inhibit the microinflammatory state of the whole body, and delay the deterioration of renal function. Proteinuria and changes in glomerular filtration membrane permeability in DN patients are closely related to vascular endothelial injury caused by oxidative stress, and DN patients often exhibit damage to the antioxidant defense system and an increase in free radical products. Probiotics can exert antioxidant effects through their own antioxidant system, such as regulating signaling pathways to produce various metabolites with antioxidant activity, such as glutathione (66).

Second, we found that compared with the usual or high-salt diet, the salt restriction diet significantly improved SBP (very low-quality evidence), DBP (very low-quality evidence), CrCl (low-quality evidence), and body weight (very low-quality evidence) in DN patients. High salt intake leads to elevated blood pressure caused by high sodium intake, which increases the risk of cardiovascular events in patients with DN. People with DN can lower their blood pressure by restricting salt, and in both type 1 and type 2 diabetes, salt restriction lasting 1 week leads to lower blood pressure (7.11/3.13 mmHg in type 1 diabetes patients and 6.90/2.87 mmHg in type 2 diabetes patients) (67). Current nutritional guidelines for patients with DN consistently recommend limiting dietary sodium intake to < 1.5 to 2.3 g/d (5 g NaCl). However, too low of a sodium intake may reduce insulin sensitivity and is not conducive to glucose homeostasis (68).

Third, the present umbrella review showed that supplementation with soy isoflavones significantly improved BUN (low-quality evidence), FBG (low-quality evidence), total cholesterol (TC) (low-quality evidence), LDL-C (low-quality evidence) and 24-hour urine protein (very low-quality evidence) in DN patients compared with no supplementation with soy isoflavones. Studies have shown that soy foods can regulate blood lipid metabolism in the body to reduce low-density lipoprotein levels and increase high-density lipoprotein levels. Moreover, plant sterols contained in soybeans can competitively inhibit the body’s cholesterol synthesis and reduce serum cholesterol levels (69). To improve kidney function, soy foods can reduce 24-h urinary protein levels. Replacing animal protein with a portion of soy protein in the diet does not adversely affect kidney function but also improves kidney hemodynamic function and reduces the elimination of urinary protein (5). Soybean protein itself is a high-quality protein and has a relatively high raw price. After the digestibility of soybean food is significantly improved, soybean protein and animal protein play the same nutritional role. Moreover, soy protein is lower in fat than animal protein is, which helps people with diabetes control the total calories in their diet and reduce the intake of too much fat, especially saturated fat, due to the consumption of human animal protein (69). More importantly, the unique nutrients of soy protein contribute to the stability of blood sugar and blood lipids in diabetic patients and can also remove excess free radicals in diabetic patients, reduce oxidative stress in the body, reduce the attack of glycoylation end products on the body’s target organs, and prevent complications (70).

However, the effect of a low-protein diet on DN has been controversial. The basic principle of low-protein diet therapy is to reverse glomerular filtration and reduce uremic symptoms. Studies on patients with chronic kidney disease and advanced DN have shown that a low-protein diet can lead to malnutrition, which is a risk factor for mortality from this disease (71). Therefore, the beneficial effect of a low-protein diet on renal prognosis may be offset by the malnutrition of the treatment itself, and more importantly, a low-protein diet may increase the mortality of DN patients (72). The results of this study showed that a low-protein diet was not significantly associated with improved kidney function in patients with DN. Although these results do not completely negate other potential benefits of a low-protein diet for DN patients, the benefits of a low-protein diet on renal function are not significant (71). Urinary tract infection is also one of the common complications in patients with DN (73, 74). However, the existing studies have not reported a significantly effective dietary intervention that can reduce the risk of urinary tract infection in patients with DN. The study by Chen et al. (73) found that vegetarianism was a protective factor for urinary tract infections, but the protective effect was not significant in the subgroup of patients with diabetes. In addition, Zaragoza-Marti et al. (75) believe that the Mediterranean diet can significantly reduce the risk of gestational diabetes and urinary tract infections, but there are no data on the effect of the Mediterranean diet on the development of urinary tract infections in diabetic patients. In addition, this study revealed that nutritional supplements such as CoQ10, dietary polyphenols and ketoanalog can effectively improve the clinical outcomes of DN patients, but the quality of evidence is low.

## Limitations and strengths

This study has several limitations. First, we searched only English language databases, and studies in other languages were excluded, which may lead to potential bias. Second, only published data were extracted, and unpublished or forthcoming evidence-based evidence was ignored. Third, this study directly extracted and analyzed existing data from systematic reviews and meta-analyses, and data from those original studies not included in systematic reviews and meta-analyses were not included. Despite these acknowledged limitations, this umbrella review provides the first comprehensive documentation of the existing evidence from prior meta-analyses on the effects of dietary intervention on DN. This umbrella review evaluated the advantages and disadvantages of existing evidence-based medicine through a systematic review and meta-analyses of the effects of dietary intervention on DN. This review helps to elucidate potential dietary management strategies for the prevention and treatment of DN in a more comprehensive way from multiple dimensions, provides a theoretical basis for the development of more clinically effective prevention and control measures for DN, and provides directions for further clinical research. This study employed rigorous systematic methodologies. Two independent authors conducted the literature searches, selected the studies, and extracted the data. When sufficient data were available, we reanalyzed the RR, WMD, or SMD using 95% CIs with random or fixed effects models. We thoroughly assessed heterogeneity and publication bias for the inclusion of each meta-analysis. Additionally, we utilized three established approaches, namely, the AMSTAR, GRADE and evidence classification criteria, to appraise the methodological quality and evidence classification of each risk factor. This comprehensive evaluation enabled us to assess our confidence in the provided estimates.

## Conclusion

The present umbrella review extracted 9 unique dietary interventions and 55 corresponding outcomes in meta-analyses, including 34 significantly associated outcomes and 21 nonsignificantly associated outcomes. All outcomes were classified as IV or NS (nonsignificant), and only five dietary interventions were rated as moderate-quality evidence. The results of this umbrella review showed that dietary interventions such as probiotics, a salt restriction diet, vitamin D, soy isoflavone, CoQ10, ketoanalog, dietary polyphenols, antioxidant vitamins, and low-protein diets could effectively delay the development of DN to some extent. The findings in this paper can aid in the development of better prevention and treatment measures to reduce the incidence of DN, delay its progression, and reduce the burden of DN-related disease worldwide.

## Data availability statement

The original contributions presented in the study are included in the article/**Supplementary Material**. Further inquiries can be directed to the corresponding authors.

## Author contributions

LC: Conceptualization, Data curation, Formal analysis, Funding acquisition, Investigation, Methodology, Project administration, Resources, Software, Supervision, Validation, Visualization, Writing – original draft, Writing – review & editing. YH: Data curation, Formal analysis, Writing – review & editing. XL: Data curation, Formal analysis, Investigation, Software, Writing – review & editing. DC: Conceptualization, Methodology, Project administration, Supervision, Validation, Writing – review & editing. FL: Conceptualization, Methodology, Project administration, Resources, Supervision, Validation, Writing – review & editing.
