# Supplementary material for: Effects of dietary intervention on diabetic nephropathy: an umbrella review of systematic reviews and meta-analyses of randomized controlled trials
Source: Front Endocrinol (Lausanne). 2024 Apr 29;15:1385872. doi: 10.3389/fendo.2024.1385872 (PMC11089238; doi:10.3389/fendo.2024.1385872)
Supplement: Supplementary file 2 [file DataSheet_2.docx]

| Supplementary Table S2. GRADE classification of quality of evidence. | | | | | | | | | | | | | | | | |
| --- | --- | --- | --- | --- | --- | --- | --- | --- | --- | --- | --- | --- | --- | --- | --- | --- |
| Dietary intervention | Control | Outcomes | Study | No. of  studies | RCT | Cohort | Case-control/ cross-sectional | Risk of  bias | Inconsistency | Indirectness | Imprecision | Publication bias | Plausible confounding | Magnitude of  effect | Dose-response gradient | Quality |
| Low protein diets | Usual diet | Change in UAER | Jiang 2023 | 2 | 2 | 0 | 0 | serious  risk | no serious  inconsistency | no serious  indirectness | serious  imprecision | strongly suspected | would not  reduce effect | no | no | very low |
| Probiotics | Usual care | Change in Scr | Dai 2022 | 6 | 6 | 0 | 0 | no serious  risk | serious  inconsistency | no serious  indirectness | serious  imprecision | undetected | would not  reduce effect | no | no | low |
| Probiotics | Usual care | Change in BUN | Dai 2022 | 5 | 5 | 0 | 0 | no serious  risk | no serious  inconsistency | no serious  indirectness | serious  imprecision | undetected | would not  reduce effect | no | no | moderate |
| Probiotics | Usual care | Change in UACR | Dai 2022 | 2 | 2 | 0 | 0 | no serious  risk | serious  inconsistency | no serious  indirectness | serious  imprecision | NA | would not  reduce effect | no | no | low |
| Probiotics | Usual care | Change in FBG | Dai 2022 | 5 | 5 | 0 | 0 | no serious  risk | no serious  inconsistency | no serious  indirectness | serious  imprecision | strongly suspected | would not  reduce effect | no | no | low |
| Probiotics | Usual care | Change in HbA1c | Dai 2022 | 4 | 4 | 0 | 0 | no serious  risk | no serious  inconsistency | no serious  indirectness | serious  imprecision | strongly suspected | would not  reduce effect | no | no | low |
| Probiotics | Usual care | Change in TC | Dai 2022 | 5 | 5 | 0 | 0 | no serious  risk | no serious  inconsistency | no serious  indirectness | serious  imprecision | undetected | would not  reduce effect | no | no | moderate |
| Probiotics | Usual care | Change in LDL-C | Dai 2022 | 5 | 5 | 0 | 0 | no serious  risk | no serious  inconsistency | no serious  indirectness | serious  imprecision | undetected | would not  reduce effect | no | no | moderate |
| Probiotics | Usual care | Change in HDL-C | Dai 2022 | 5 | 5 | 0 | 0 | no serious  risk | serious  inconsistency | no serious  indirectness | serious  imprecision | undetected | would not  reduce effect | no | no | low |
| Salt restriction diet | Usual or high salt diet | Change in SBP | Hodson 2023 | 12 | 12 | 0 | 0 | serious  risk | serious  inconsistency | no serious  indirectness | serious  imprecision | undetected | would not  reduce effect | no | no | very low |
| Salt restriction diet | Usual or high salt diet | Change in DBP | Hodson 2023 | 12 | 12 | 0 | 0 | serious  risk | serious  inconsistency | no serious  indirectness | serious  imprecision | undetected | would not  reduce effect | no | no | very low |
| Salt restriction diet | Usual or high salt diet | Change in CrCl | Hodson 2023 | 7 | 7 | 0 | 0 | serious  risk | no serious  inconsistency | no serious  indirectness | serious  imprecision | undetected | would not  reduce effect | no | no | low |
| Salt restriction diet | Usual or high salt diet | Change in body weight | Hodson 2023 | 12 | 12 | 0 | 0 | serious  risk | serious  inconsistency | no serious  indirectness | serious  imprecision | undetected | would not  reduce effect | no | no | very low |
| Vitamin D | Placebo | Change in UACR | He 2022 | 6 | 6 | 0 | 0 | no serious  risk | no serious  inconsistency | no serious  indirectness | serious  imprecision | strongly suspected | would not  reduce effect | no | no | low |
| Vitamin D | Placebo | Change in UAER | He 2022 | 4 | 4 | 0 | 0 | no serious  risk | serious  inconsistency | no serious  indirectness | serious  imprecision | strongly suspected | would not  reduce effect | no | no | very low |
| Vitamin D | Without vitamin D or placebo | Change in 24-hour urine protein | Wang 2019 | 11 | 11 | 0 | 0 | no serious  risk | serious  inconsistency | no serious  indirectness | serious  imprecision | strongly suspected | would not  reduce effect | no | no | very low |
| Soy isoflavone | Without soy isoflavone | Change in 24-hour urine protein | Wang 2021 | 6 | 6 | 0 | 0 | serious  risk | serious  inconsistency | no serious  indirectness | serious  imprecision | NA | would not  reduce effect | no | no | very low |
| Soy isoflavone | Without soy isoflavone | Change in BUN | Wang 2021 | 7 | 7 | 0 | 0 | serious  risk | no serious  inconsistency | no serious  indirectness | serious  imprecision | NA | would not  reduce effect | no | no | low |
| Soy isoflavone | Without soy isoflavone | Change in FBG | Wang 2021 | 5 | 5 | 0 | 0 | serious  risk | no serious  inconsistency | no serious  indirectness | serious  imprecision | NA | would not  reduce effect | no | no | low |
| Soy isoflavone | Without soy isoflavone | Change in TC | Wang 2021 | 8 | 8 | 0 | 0 | serious  risk | no serious  inconsistency | no serious  indirectness | serious  imprecision | NA | would not  reduce effect | no | no | low |
| Soy isoflavone | Without soy isoflavone | Change in TG | Wang 2021 | 8 | 8 | 0 | 0 | serious  risk | no serious  inconsistency | no serious  indirectness | serious  imprecision | NA | would not  reduce effect | no | no | low |
| Soy isoflavone | Without soy isoflavone | Change in LDL-C | Wang 2021 | 7 | 7 | 0 | 0 | serious  risk | no serious  inconsistency | no serious  indirectness | serious  imprecision | NA | would not  reduce effect | no | no | low |
| CoQ10 | Placebo | Change in FBG | Zhang 2019 | 3 | 3 | 0 | 0 | no serious  risk | serious  inconsistency | no serious  indirectness | serious  imprecision | strongly suspected | would not  reduce effect | no | no | very low |
| CoQ10 | Placebo | Change in HbA1c | Zhang 2019 | 3 | 3 | 0 | 0 | no serious  risk | serious  inconsistency | no serious  indirectness | serious  imprecision | undetected | would not  reduce effect | no | no | low |
| CoQ10 | Placebo | Change in TC | Zhang 2019 | 3 | 3 | 0 | 0 | no serious  risk | serious  inconsistency | no serious  indirectness | serious  imprecision | undetected | would not  reduce effect | no | no | low |
| CoQ10 | Placebo | Change in HDL-C | Zhang 2019 | 3 | 3 | 0 | 0 | no serious  risk | serious  inconsistency | no serious  indirectness | serious  imprecision | undetected | would not  reduce effect | no | no | low |
| Ketoanalogue | Without ketoanalogue | Change in 24-hour urine protein | Bellizzi 2022 | 5 | 5 | 0 | 0 | serious  risk | serious  inconsistency | no serious  indirectness | serious  imprecision | undetected | would not  reduce effect | no | no | very low |
| Ketoanalogue | Without ketoanalogue | Change in FBG | Bellizzi 2022 | 7 | 7 | 0 | 0 | serious  risk | serious  inconsistency | no serious  indirectness | serious  imprecision | undetected | would not  reduce effect | no | no | very low |
| Dietary polyphenols | Without polyphenols or placebo | Change in HbA1c | Macena 2022 | 7 | 7 | 0 | 0 | serious  risk | no serious  inconsistency | no serious  indirectness | serious  imprecision | NA | would not  reduce effect | no | no | low |
| Dietary polyphenols | Without polyphenols or placebo | Change in GFR | Macena 2022 | 7 | 7 | 0 | 0 | serious  risk | serious  inconsistency | no serious  indirectness | serious  imprecision | NA | would not  reduce effect | no | no | very low |
| Dietary polyphenols | Without polyphenols or placebo | Change in 24-hour urine protein | Macena 2022 | 5 | 5 | 0 | 0 | serious  risk | serious  inconsistency | no serious  indirectness | serious  imprecision | NA | would not  reduce effect | no | no | very low |
| Antioxidant vitamins | Placebo | Change in Scr | Chen 2020 | 3 | 3 | 0 | 0 | serious  risk | no serious  inconsistency | no serious  indirectness | serious  imprecision | NA | would not  reduce effect | no | no | low |
| Antioxidant vitamins | Placebo | Change in SBP | Chen 2020 | 5 | 5 | 0 | 0 | serious  risk | no serious  inconsistency | no serious  indirectness | serious  imprecision | NA | would not  reduce effect | no | no | low |
| Antioxidant vitamins | Placebo | Change in HbA1c | Chen 2020 | 6 | 6 | 0 | 0 | serious  risk | no serious  inconsistency | no serious  indirectness | serious  imprecision | NA | would not  reduce effect | no | no | low |
| Low protein diets | Usual diet | All-cause mortality | Jiang 2023 | 5 | 5 | 0 | 0 | no serious  risk | no serious  inconsistency | no serious  indirectness | serious  imprecision | strongly suspected | would not  reduce effect | no | no | low |
| Low protein diets | Usual diet | Renal failure | Jiang 2023 | 4 | 4 | 0 | 0 | no serious  risk | no serious  inconsistency | no serious  indirectness | serious  imprecision | strongly suspected | would not  reduce effect | no | no | low |
| Low protein diets | Usual diet | Change in GFR | Jiang 2023 | 7 | 7 | 0 | 0 | serious  risk | serious  inconsistency | no serious  indirectness | serious  imprecision | strongly suspected | would not  reduce effect | no | no | very low |
| Low protein diets | Usual diet | Change in CrCl | Jiang 2023 | 3 | 3 | 0 | 0 | serious  risk | serious  inconsistency | no serious  indirectness | serious  imprecision | strongly suspected | would not  reduce effect | no | no | very low |
| Low protein diets | Usual diet | Change in 24-hour urinary albumin excretion | Jiang 2023 | 2 | 2 | 0 | 0 | serious  risk | no serious  inconsistency | no serious  indirectness | serious  imprecision | strongly suspected | would not  reduce effect | no | no | very low |
| Probiotics | Placebo | Change in GFR | Dai 2022 | 5 | 5 | 0 | 0 | no serious  risk | serious  inconsistency | no serious  indirectness | serious  imprecision | undetected | would not  reduce effect | no | no | low |
| Salt restriction diet | Usual or high salt diet | Change in GFR | Hodson 2023 | 12 | 12 | 0 | 0 | serious  risk | no serious  inconsistency | no serious  indirectness | serious  imprecision | undetected | would not  reduce effect | no | no | low |
| Salt restriction diet | Usual or high salt diet | Change in HbA1c | Hodson 2023 | 6 | 6 | 0 | 0 | serious  risk | serious  inconsistency | no serious  indirectness | serious  imprecision | undetected | would not  reduce effect | no | no | very low |
| Vitamin D | Without vitamin D or placebo | Change in Scr | Wang 2019 | 9 | 9 | 0 | 0 | no serious  risk | no serious  inconsistency | no serious  indirectness | serious  imprecision | strongly suspected | would not  reduce effect | no | no | low |
| Vitamin D | Without vitamin D or placebo | Change in GFR | Wang 2019 | 4 | 4 | 0 | 0 | no serious  risk | no serious  inconsistency | no serious  indirectness | serious  imprecision | strongly suspected | would not  reduce effect | no | no | low |
| Vitamin D | Without vitamin D or placebo | Change in HbA1c | Wang 2019 | 10 | 10 | 0 | 0 | no serious  risk | no serious  inconsistency | no serious  indirectness | serious  imprecision | undetected | would not  reduce effect | no | no | moderate |
| Vitamin D | Without vitamin D or placebo | Change in FBG | Wang 2019 | 3 | 3 | 0 | 0 | no serious  risk | no serious  inconsistency | no serious  indirectness | serious  imprecision | strongly suspected | would not  reduce effect | no | no | low |
| Soy isoflavone | Without soy isoflavone | Change in body weight | Wang 2021 | 6 | 6 | 0 | 0 | serious  risk | no serious  inconsistency | no serious  indirectness | serious  imprecision | NA | would not  reduce effect | no | no | low |
| Soy isoflavone | Without soy isoflavone | Change in Scr | Wang 2021 | 7 | 7 | 0 | 0 | serious  risk | no serious  inconsistency | no serious  indirectness | serious  imprecision | NA | would not  reduce effect | no | no | low |
| Soy isoflavone | Without soy isoflavone | Change in CrCl | Wang 2021 | 3 | 3 | 0 | 0 | serious  risk | no serious  inconsistency | no serious  indirectness | serious  imprecision | NA | would not  reduce effect | no | no | low |
| Soy isoflavone | Without soy isoflavone | Change in GFR | Wang 2021 | 6 | 6 | 0 | 0 | serious  risk | no serious  inconsistency | no serious  indirectness | serious  imprecision | NA | would not  reduce effect | no | no | low |
| Soy isoflavone | Without soy isoflavone | Change in HDL-C | Wang 2021 | 7 | 7 | 0 | 0 | serious  risk | no serious  inconsistency | no serious  indirectness | serious  imprecision | NA | would not  reduce effect | no | no | low |
| CoQ10 | Placebo | Change in LDL-C | Zhang 2019 | 3 | 3 | 0 | 0 | no serious  risk | no serious  inconsistency | no serious  indirectness | serious  imprecision | undetected | would not  reduce effect | no | no | moderate |
| Ketoanalogue | Without ketoanalogue | Change in GFR | Bellizzi 2022 | 4 | 4 | 0 | 0 | serious  risk | serious  inconsistency | no serious  indirectness | serious  imprecision | undetected | would not  reduce effect | no | no | very low |
| Antioxidant vitamins | Placebo | Change in DBP | Chen 2020 | 5 | 5 | 0 | 0 | serious  risk | no serious  inconsistency | no serious  indirectness | serious  imprecision | NA | would not  reduce effect | no | no | low |
| Antioxidant vitamins | Placebo | Change in FBG | Chen 2020 | 5 | 5 | 0 | 0 | serious  risk | no serious  inconsistency | no serious  indirectness | serious  imprecision | NA | would not  reduce effect | no | no | low |
| UAER, Urinary albumin excretion rate; Scr, serum creatinine; BUN, blood urea nitrogen; UACR, urinary albumin creatinine ratio; FBG, fasting blood-glucose; TC, total cholesterol; LDL-C, low-density lipoprotein cholesterol; HDL-C, high-density lipoprotein cholesterol; SBP, systolic blood pressure; DBP, diastolic blood pressure; CoQ10, coenzyme Q10; GFR, glomerular filtration rate; CrCl, creatinine clearance rate; NA, not available. | | | | | | | | | | | | | | | | |
